# Targeting Nonsense: Optimization of 1,2,4-Oxadiazole TRIDs to Rescue CFTR Expression and Functionality in Cystic Fibrosis Cell Model Systems

**DOI:** 10.3390/ijms21176420

**Published:** 2020-09-03

**Authors:** Ivana Pibiri, Raffaella Melfi, Marco Tutone, Aldo Di Leonardo, Andrea Pace, Laura Lentini

**Affiliations:** 1 Dipartimento di Scienze e Tecnologie Biologiche, Chimiche e Farmaceutiche (STEBICEF), Università degli Studi di Palermo, Viale delle Scienze Ed. 16-17, 90128 Palermo, Italy; raffaella.melfi@unipa.it (R.M.); marco.tutone@unipa.it (M.T.); aldo.dileonardo@unipa.it (A.D.L.); andrea.pace@unipa.it (A.P.); 2 Centro di OncoBiologia Sperimentale (COBS), via San Lorenzo Colli, 90145 Palermo, Italy

**Keywords:** premature termination codon, nonsense mutation, genetic disorder, translational readthrough inducing drugs, oxadiazoles

## Abstract

Cystic fibrosis (CF) patients develop a severe form of the disease when the cystic fibrosis transmembrane conductance regulator (CFTR) gene is affected by nonsense mutations. Nonsense mutations are responsible for the presence of a premature termination codon (PTC) in the mRNA, creating a lack of functional protein. In this context, translational readthrough-inducing drugs (TRIDs) represent a promising approach to correct the basic defect caused by PTCs. By using computational optimization and biological screening, we identified three new small molecules showing high readthrough activity. The activity of these compounds has been verified by evaluating CFTR expression and functionality after treatment with the selected molecules in cells expressing nonsense–CFTR–mRNA. Additionally, the channel functionality was measured by the halide sensitive yellow fluorescent protein (YFP) quenching assay. All three of the new TRIDs displayed high readthrough activity and low toxicity and can be considered for further evaluation as a therapeutic approach toward the second major cause of CF.

## 1. Introduction

Protein synthesis is a crucial process for life; therefore, gene mutations can seriously compromise the quality of life and even life expectancy.

A point mutation in the gene can result in a silent, missense, or nonsense mutation. In the fortunate case of a silent mutation, there are no effects on the amino acid sequence of the resulting protein. A missense mutation, which alters the primary structure, can cause protein malfunctioning due to incorrect folding or different conformations in the active site of a specific functional protein [1].

In the worst case, when a nonsense mutation is present in the gene producing a premature termination codon in the transcript, the protein production is compromised. A surveillance mechanism named NMD (nonsense-mediated decay) intervenes to destroy aberrant transcripts, and the small amount of leftover mRNA bearing the PTC will produce truncated polypeptides that, in turn, will be degraded as aberrant protein [2].

When a PTC occurs in the transcript, a basal readthrough partially restores protein synthesis to a very small extent, although this is too low to make an improvement in the disease phenotype [3].

**Figure 1:**
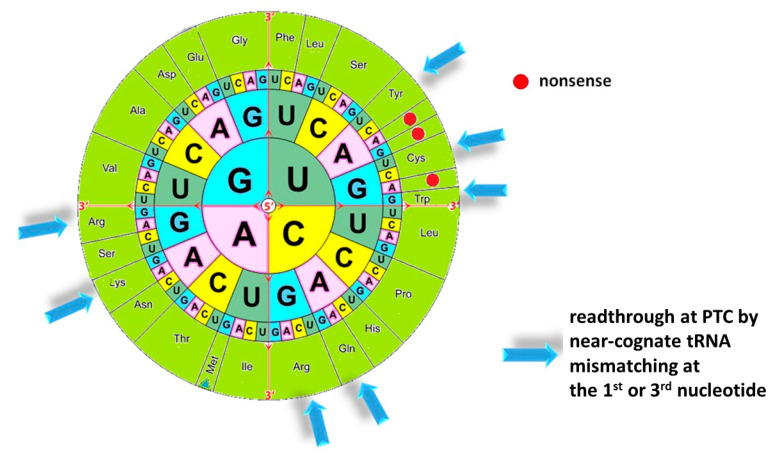
Codons language and most frequent misreadings for premature termination codon (PTC)’s readthrough.

If compared to basal readthrough, drug-induced readthrough allows for the restoration of protein synthesis to a wider extent [4]. Both basal and drug-induced readthrough of PTCs are known to occur by a misreading of the PTC by a near-cognate tRNA [5,6,7,8]. In particular, it has been suggested that a tRNA carrying tryptophan, cysteine, or arginine misreads the third base (A/G for tryptophan; A/U,C for cysteine) or the first base (U/C for arginine) of the UGA premature termination codon. The same kind of misreading could justify the insertion of glutamine, tyrosine, or lysine during the readthrough of the other two PTCs (UAG and UAA), as sketched in a genetic language translation roulette in Figure 1 [3,8].

The readthrough process depends on the kind of PTC, its location in the gene, and the surrounding mRNA sequence. Its efficiency is affected by the nucleotide immediately downstream of the PTC; moreover, the role of ancillary bases upstream of the PTC has recently been noted in the literature regarding drug-induced readthrough [7,8,9].

The amount of protein rescued by readthrough can be raised by NMD inhibition, as reported in many cases [10,11,12,13,14].

The production of a functional protein is guaranteed when the readthrough causes the insertion of the wild-type amino acid in the correspondence of the PTC. Alternatively, the readthrough can also skip the nonsense and produce the effect of a missense mutation, as widely discussed [15,16].

Nonsense mutations are found in about 11% of genetic disorders such as cystic fibrosis (CF) [17], Duchenne muscular dystrophy (DMD) [18], spinal muscular atrophy [19,20], neurofibromatosis [21], retinitis pigmentosa [22,23,24], lysosomal storage disease [13,25,26], Rett syndrome [27], Shwachman–Diamond syndrome [28,29], and Usher’s syndrome (USH) [30]. In this context and despite its limits, the translational readthrough of PTCs can represent a valuable pharmaceutical approach to target the specific genetic defect caused by nonsense mutations.

Among genetic disorders, cystic fibrosis (CF) is not a rare disease as it involves more than 100,000 people worldwide and affects approximately 36,000 individuals in the European Union (EU), while 1000 new cases of CF are diagnosed each year in the US [31].

The cystic fibrosis transmembrane conductance regulator (CFTR) gene encodes for a channel protein, which is fundamental for cellular homeostasis. CFTR starts its biogenesis by co-translational insertion into the membrane of the endoplasmic reticulum and, by following the folding of the cytosolic domains, takes on its native fully folded compact structure. Then, CFTR is modified at the Golgi apparatus and delivered to the plasmatic membrane. It works as a cAMP- and phosphorylation-regulated chloride/bicarbonate channel [32]. Both its biogenesis and functional chloride secretion and hydration determine its overall activity and, thus, contribute to the fine modulation of the homeostasis of the epithelial surfaces [32].

About 2000 different mutations have been described as being involved in the onset of the disease [33], some of which are more common, such as the deletion of the triplet encoding for phenylalanine 508 (∆F508) or the missense mutation, which substitutes glycine 551 with aspartic acid (G551D).

In addition to addressing the disease symptomatically, a personalized medicinal approach targeting the specific protein defect has also been used recently in many studies in the CF field [34,35,36,37,38].

Recently, a pharmacological treatment that combines three different drugs (elexacaftor/tezacaftor/ivacaftor), which act synergistically, has been approved to overcome the ∆F508 CFTR defect. On the other hand, however, nonsense mutations, which recur in 10–15% of the CF cases worldwide, are still lacking an effective pharmacological approach.

**Figure 2:**
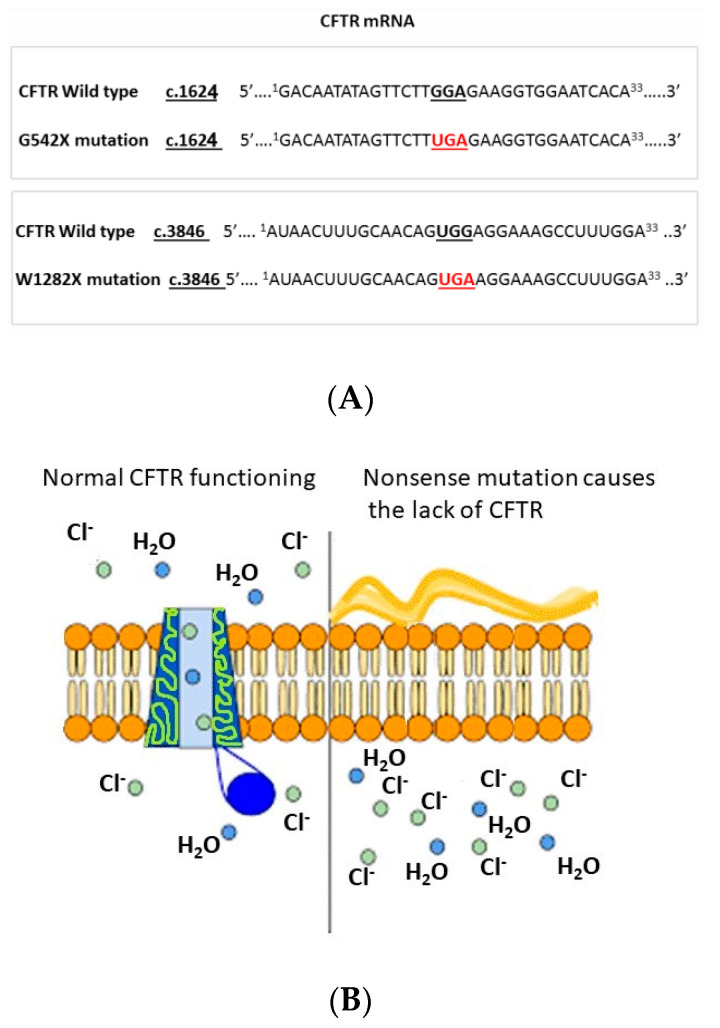
Illustrations of cystic fibrosis transmembrane conductance regulator (CFTR) nonsense mutations and their effect. (**A**) CFTR mRNA sequence of the stop codon G542X and W1282X (in red the PTC UGA codons). (**B**) CFTR localization and effect of the nonsense mutation on the CFTR gene, impairing cell homeostasis and causing the accumulation of mucus and the following infection.

Despite the significant efforts devoted to finding a cure for CF, there is no current therapy for the nonsense mutations of the CFTR gene that cause the lack of CFTR protein and are responsible for a more severe form of the disease (Figure 2A,B) [39].

A potential therapeutic approach is represented by the development of drugs inducing the readthrough of nonsense mutations. By promoting a selective bypass of the PTC, the readthrough approach would restore the expression of the CFTR protein and, once functionality is rescued to a sufficient extent, improve cell homeostasis.

**Figure 3:**
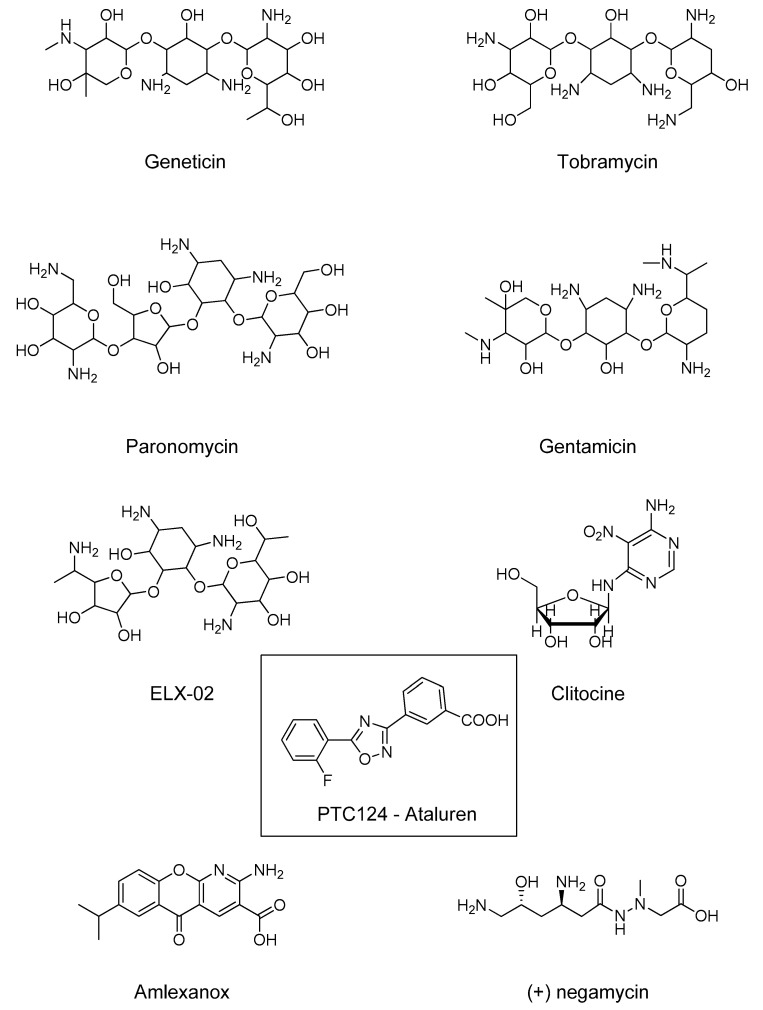
Different structures of proposed translational readthrough-inducing drugs (TRIDs).

The first drugs tested as translational readthrough-inducing drugs (TRIDs) were aminoglycosides (e.g., gentamicin, tobramycin, paromomycin, geneticin; see Figure 3), which have been shown to suppress the normal proof-reading function of the ribosome [40,41]. These drugs, which were already known for their antibiotic activity, induce the readthrough of the PTCs continuing the translation process [42]. Unfortunately, the chronic use of high dosages of aminoglycosides is associated with severe side-effects such as renal, auditory, and vestibular toxicities [43], which is what inhibited their pharmaceutical implementation. Nevertheless, a new aminoglycoside, showing potential activity as a TRID and reduced toxicity, has been tested in healthy subjects and is currently proceeding further in clinical trials [44].

To overcome the aminoglycoside toxicity, different molecules such as (+)-negamicine [45], amlexanox [25], clitocine [46], and PTC124 (also known as ataluren) [47] (Figure 3) have been proposed as readthrough promoters. Among these, ataluren was able to promote the readthrough of PTCs, particularly UGA, without affecting the normal termination codons [47], thus being a good candidate for developing a therapy for nonsense mutations [48].

Phase I and II clinical trials, including healthy subjects and CF and Duchenne muscular dystrophy (DMD) patients, have evaluated the long-term safety of ataluren.

Trials showed improvement of some markers of CFTR function in CF patients; however, in a new phase III trial, sweat chloride levels and the nasal potential difference were not improved [49]. Due to conflicting results, the phase III clinical study of PTC124 was suspended in April 2017 for CF patients, while the drug has been registered with the regulatory authorities and is currently in use for DMD patients [49,50]. Specifically, during the trial with CF patients, reports have shown that there is a competition between ataluren and tobramycin (which interacts at the level of the ribosome) when administered simultaneously [49].

Our previous studies on ataluren and its analogs aimed to look for alternative molecules and to understand the effective biological target and mechanism of action of TRIDs [9,10,51,52,53,54].

We hypothesized that ataluren could interact differently with the PTCs, depending on their localization in the CFTR gene (e.g., G542X, R1162X, W1282X) and on the genetic context [9].

Our research has focused on oxadiazoles, as they are widely recognized as lead compounds in medicinal chemistry [55,56,57,58,59], particularly as bioisosteric replacements for ester and amide functionalities.

The flaws in the clinical trials for ataluren, as well as the discrete toxicity evidenced in the MTT test (see Figure S1) for our recently reported lead compound NV2445 [54], pushed us to find new alternative leads.

By performing an in-silico optimization of the lead molecule NV2445 [54] using public and “in-house” compound libraries, we designed and synthesized three small molecules containing a 1,2,4-oxadiazole as the heterocyclic core and evaluated their efficacy as TRIDs.

The preliminary biological screening was performed by using the firefly luciferase reporter; the activity was confirmed by further assays evaluating the efficacy in promoting the production of a functional CFTR protein in cells engineered to express the nonsense CFTR^G542X^ or CFTR^W1282X^ mRNA. Additional tests were conducted in vitro to evaluate the toxicity of the synthesized molecules.

## 2. Results and Discussion

### 2.1. Design and Synthesis

**Figure 4:**
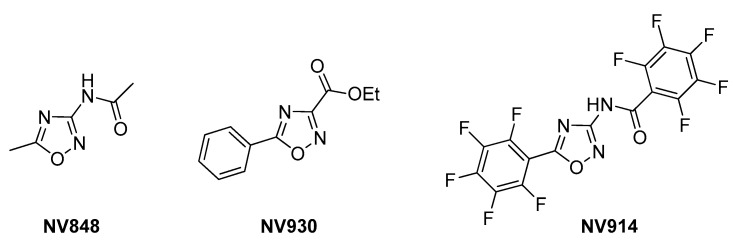
1,2,4-oxadiazoles selected structures.

The 3D-QSAR pharmacophore modeling was carried out based on our previous results [54], which allowed the identification of a validated pharmacophore model. This model was used as a query to screen public and “in-house” compound libraries. From the top 5% of retrieved hits, we selected three synthetically accessible 1,2,4-oxadiazoles (Figure 4).

Interestingly, despite the common oxadiazole core, the suggested structures presented significant differences with respect to ataluren in terms of the lack of aryl substituents (**NV848**), the lack of fluorine and acidic moieties (**NV848** and **NV930**), and the maximized fluorine content (**NV914**).

The synthesis and crystal structure of 3-acetylamino-5-methyl-1,2,4-oxadiazole, **NV848**, has been previously reported in the literature [60,61,62].

**Scheme 1:**
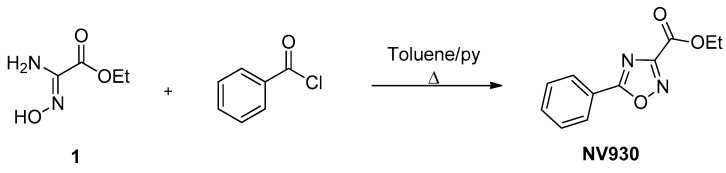
Synthesis of NV930.

Ethyl 5-phenyl-1,2,4-oxadiazole-3-carboxylate (**NV930**) was prepared by the classic amidoxime route, as illustrated in Scheme 1.

**Scheme 2:**
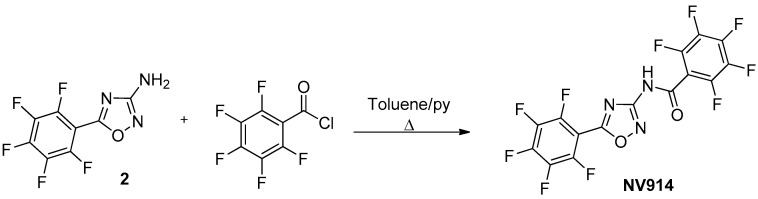
Synthesis of NV914.

2,3,4,5,6-Pentafluoro-*N*-(5-(perfluorophenyl)-1,2,4-oxadiazol-3-yl)benzamide (**NV914**) was prepared by acylation of the amine **2** [60,61,62] with perfluorobenzoyl chloride, as reported in Scheme 2.

All the synthesized compounds were purified by preparative flash chromatography and crystallization, analyzed by spectroscopic techniques, and displayed an appropriate purity grade for the biological assays.

### 2.2. Testing Cell Proliferation and Viability

Before testing the activity of the compounds as readthrough promoters, we tested their toxicity. Cell viability was assessed by the MTT assay after 24 and 72 h of exposition to **NV848**, **NV91**, and **NV930**. The assay showed that cell viability was already affected after 24 h of treatment with either PTC124 (48 μM) or NV2445 (48 μM), used as control. In contrast, **NV848**, **NV914**, and **NV930** molecules did not show altered cell viability or proliferation (Figure S1) at the same time and concentration points. A partial cytotoxic effect was observed at 72 h, especially after the addition of **NV930** at the doses of 24 and 48 μM.

### 2.3. Experimental Screening of the Readthrough Activity by Luciferase Assays, Immunofluorescence, and Western Blot Analyses

To ascertain the efficacy of the newly synthesized small molecules in promoting the readthrough of premature termination codons, we used the FLuc cell-based assay [52,53]. Although it is not usually reliable as a quantitative assessment, this screening is easy and useful to find potential readthrough promoters. HeLa cells were transiently transfected with the plasmids pFLuc-WT (positive control) and pFLuc-opal (UGA stop mutation) [63]. After 24 h, the new molecules were added to the cells and, finally, 48 h after the transfection, FLuc gene expression was measured by luminescence.

**Figure 5:**
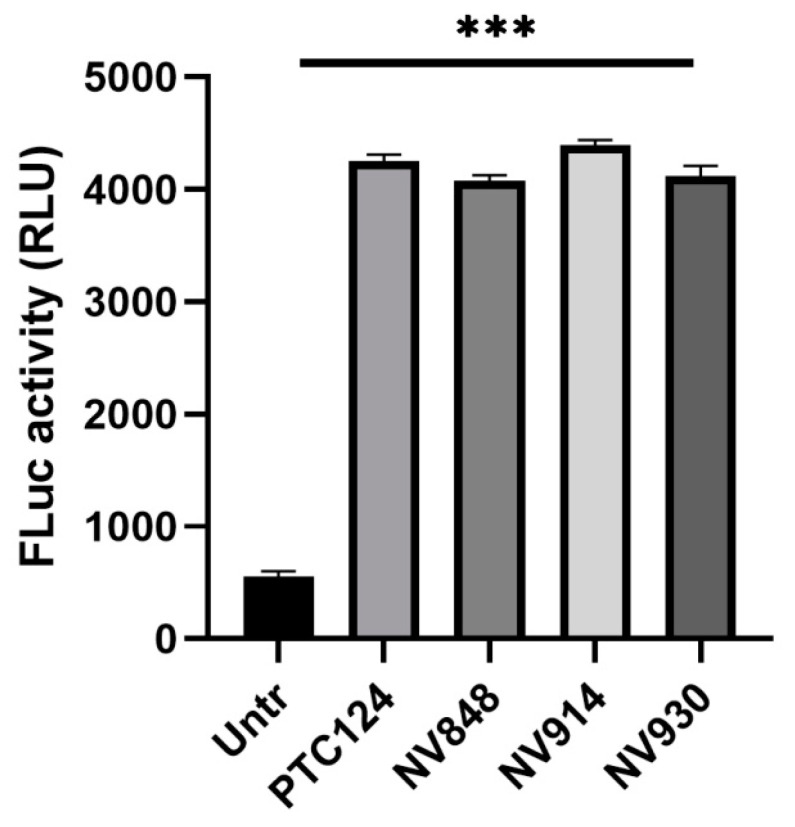
The newly synthesized molecules show readthrough activity in the FLuc cell model system. Histogram shows luciferase (FLuc) activity (RLU) after 24 h of exposition to PTC124, **NV848**, **NV930**, and **NV914** (all at the concentration of 12 μM) in HeLa FLuc-opal transfected cells. Data were analyzed by GraphPad Prism 6 software and expressed as mean values ± standard error of the mean (SEM; *n* = 3). Symbol (*) represents the statistical significance of data regarding PTC124, **NV848**, **NV914**, and **NV930** versus Untr: ***, *p* < 0.001 (ANOVA with Dunnett’s posthoc test).

HeLa cells transfected with the pFluc-WT plasmid were used as a positive control of the experiment. These cells showed high levels of luciferase activity (about 5 × 10^6^ activity/relative luminescence units (RLU)). On the other hand, untreated pFluc-opal transfected HeLa cells did not show any activity (negative control). However, when pFluc-opal cells were treated with PTC124 and the three different analogs, we observed a significant increase of luciferase activity in comparison to untreated cells (Figure 5).

The readthrough activity on the CFTR mRNA was studied in Fisher rat thyroid (FRT) cells. FRT cells do not express endogenous CFRT and other endogenous cAMP-regulated Cl^−^ channels. The cells were transfected with a vector harboring the full-length CFTR cDNA (pTracer-CFTR) [64]. The vector harbored either a wild-type (WT) or a mutated CFTR cDNA in which we previously introduced one of the two stop mutations, G542X or W1282X, by site-directed mutagenesis [54].

To evaluate the readthrough activity of the three NV molecules, FRT cells transfected with the mutated cDNA were treated for 24 h with **NV848**, **NV914**, **NV930**, or PTC124 (positive control) at a 12 μM concentration, chosen for better comparison to ataluren’s reported activity [51]. The expression of the human CFTR-WT and the rescued CFTR (from CFTR^G542X^ or CFTR^W1282X^) were detected by immunofluorescence and Western blot analyses.

Dose–response activity was tested only on FRT CFTR^W1282X^, and the maximum readthrough activity was recorded at 24 μM for **NV848** and **NV930** (see Figure S2B,D). For **NV914**, the highest activity was recorded at 48 μM, with a very small increase with respect to activity at 24 μM (see Figure S2C). The experiments were performed in duplicate and allowed us to obtain a preliminary estimate of the EC50 in the range 6–16 μM (see Table S1), although further tests are needed to define the new TRIDs’ efficacy, especially for different mutations.

**Figure 6:**
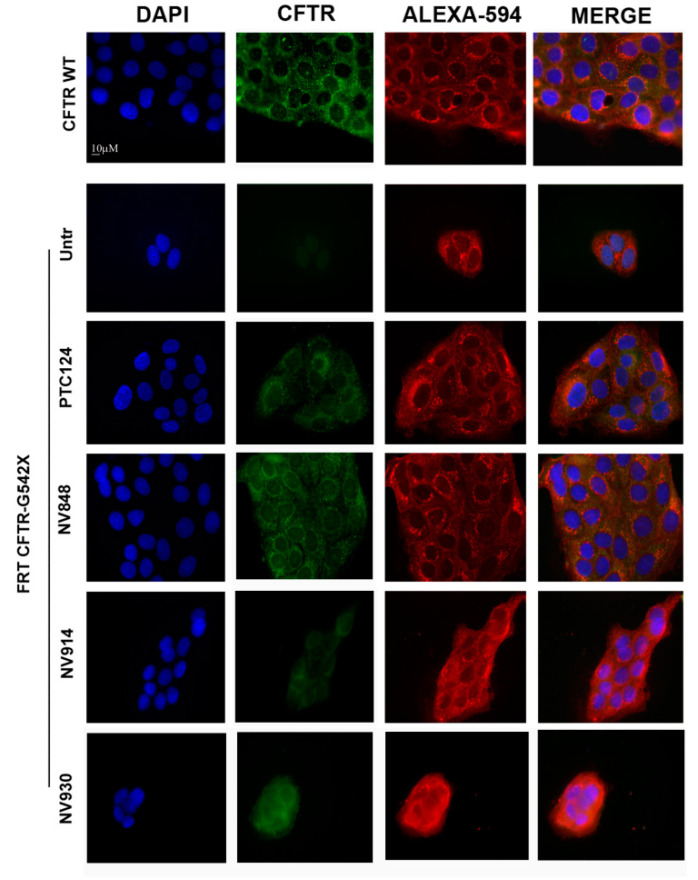
Immunofluorescence analysis of the full-length CFTR protein in wild-type (CFTR-WT) and CFTR^G542X^ Fisher rat thyroid (FRT) cells, untreated (Untr) or treated with 12 μM PTC124, **NV848**, **NV930**, or **NV914** for 24 h. CFTR protein was revealed by a specific antibody (ab570-green). Nuclei (blue) were DAPI (4′,6-diamidino-2-phenylindole)-stained and the cellular membrane was stained by wheat germ agglutinin (WGA)-Alexa 594 (red).

**Figure 7:**
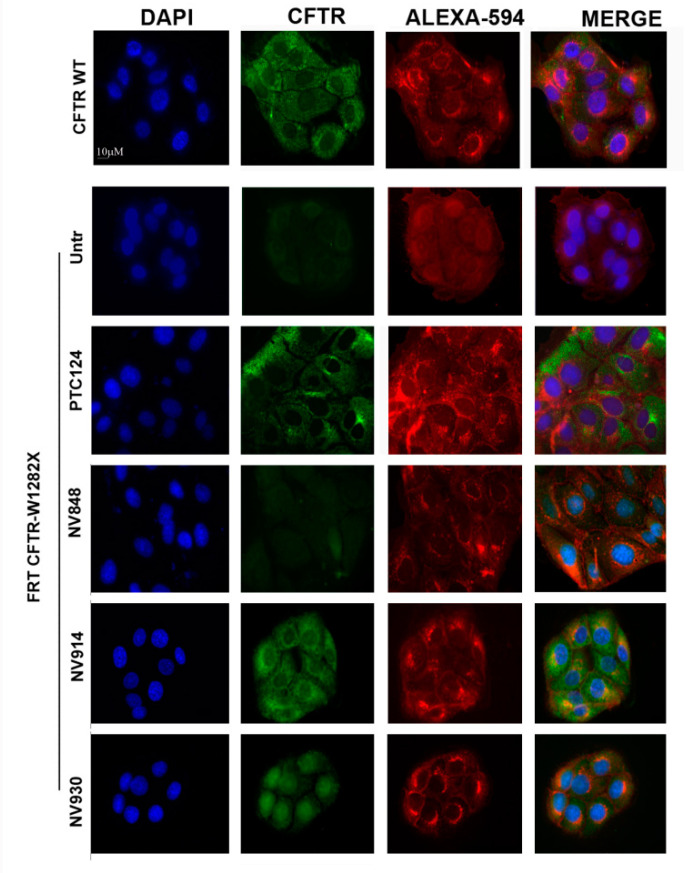
Immunofluorescence analysis of the full-length CFTR protein in wild-type (CFTR-WT) and CFTR^W1282X^ FRT cells, untreated (Untr) or treated with 12 μM PTC124, **NV848**, **NV930**, or **NV914** for 24 h. CFTR protein was revealed by a specific antibody (ab570-green). Nuclei (blue) were DAPI-stained and the cellular membrane was stained by WGA-Alexa 594 (red).

**Figure 8:**
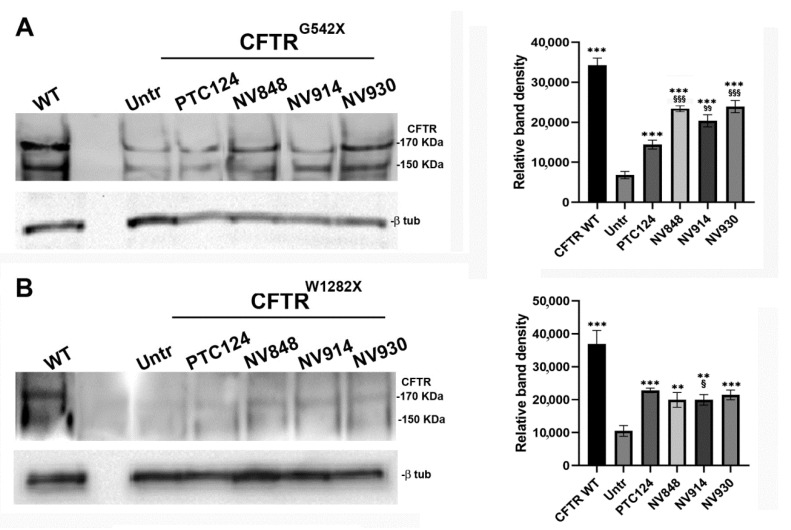
Western blot analysis of the CFTR protein in wild-type (WT) CFTR, and CFTR^G542X^ (**A**) and CFTR^W1282X^ (**B**) FRT cells, untreated (Untr) or treated with 12 μM PTC124 (positive control), **NV848**, **NV930**, or **NV914** for 24 h; β-tubulin was used as control for the protein loading. The graphs show the quantification of the gel bands by ImageJ software. The experiment was performed in triplicate and data were normalized by β-tubulin expression, analyzed by GraphPad Prism 6 software. Data are expressed as mean values ± standard error of the mean (SEM). Symbols (* and §) represent the statistical significance of **NV848**, **NV914**, and **NV930** versus Untr (*) and versus PTC124 (§). two symbols, *p* < 0.01; three symbols, *p* < 0.001 (Student’s *t*-test and ANOVA with Dunnett’s posthoc test).

Immunofluorescence microscopy images allowed us to visualize CFTR localization at the cell membrane, in particular when the CFTR^G542X^ FRT cells were exposed to **NV848** and **NV930**, suggesting the occurrence of translational readthrough after the treatments (Figure 6). On the other hand, in CFTR^W1282X^ FRT cells, the expression of the CFTR protein was significantly evident after treatment with **NV914** and **NV930**, as shown by immunofluorescence (Figure 7). Similar results were detected and quantified by Western blot analyses (Figure 8A,B and Figure S3A,B).

### 2.4. Testing Functionality of the Recovered CFTR

While the NMD pathway affects the amount of recovered protein, the variability of tRNA insertion plays a role in the quality of the protein produced by readthrough. In this context, besides measuring the effective protein production by quantitative methods, it is fundamental to prove protein functionality after readthrough.

For this purpose, molecular and functional analyses have been conducted by halide-sensitive YFP assay to evaluate the functionality of the CFTR channel produced in FRT cells after treatment with NV848, **NV914,** and **NV930**.

**Figure 9:**
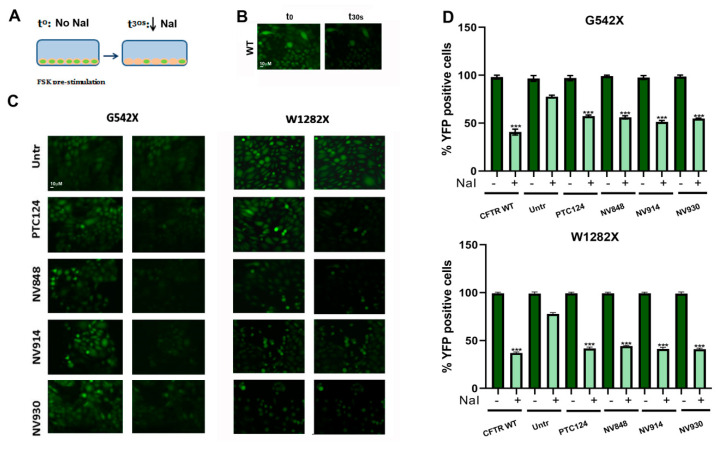
CFTR functionality in FRT cells measured by the yellow fluorescent protein (YFP) assay (pink: cells, green: YFP protein expression, blue: multiwell), (**A**) in YFP-CFTR^WT^ FRT, and (**B**) CFTR^G542X^ and CFTR^W1282X^ FRT cells, untreated or treated with 12 μM PTC124, **NV848**, **NV914**, or **NV930** for 24 h (**C**). Cells were prestimulated with 20 μM forskolin for 20 min. The experiment was performed in triplicate. Images were taken before (t^0^) and after (t^30s^) the addition of NaI. Percentages of YFP-positive cells before (− dark green bars) and after NaI addition (+ light green bars); data were analyzed by GraphPad (*n* = 3) and expressed as mean values ± standard error of the mean (SEM) (**D**). Data were analyzed comparing treated FRT cells versus untreated FRT cells by GraphPad Prism 6 software (*n* = 3). ***, *p* < 0.001 (ANOVA with Dunnett’s posthoc test).

The assay is based on the sensibility of the YFP protein to the I^-^ ion. In fact, iodide anions induce the quenching of YFP fluorescence (Figure 9A), thus evidencing the channel activity [65].

To evaluate the activity of the CFTR channel, CFTR^G542X^ and CFTR^W1282X^ FRT cells were treated for 24 h with either one of the **NV848**, **NV914**, or **NV930** compounds. After 24 h, cells were prestimulated with forskolin to activate the channel and observed by microscopy before and after the addition of NaI. Treated CFTR^G542X^ and CFTR^W1282X^, as well as WT-CFTR, FRT cells showed a significant quenching of the YFP fluorescence after the addition of iodide (Figure 9B,C), thus proving the channel functionality. Interestingly, the response to the YFP assay was different based on the stop codon undergoing the readthrough. In fact, CFTR^G542X^ FRT cells clearly showed a YFP fluorescence quenching when treated with **NV848**, **NV914**, or **NV930** (Figure 9C, left) while CFTR^W1282X^ FRT cells showed quenching of the YFP fluorescence when specially treated with **NV930** (Figure 9C, right). The same experiments were performed to measure the YFP fluorescence quenching after treatment with the three molecules in a fluorescent microplate reader and data are illustrated in Figure 9D as the percentage of fluorescent cells before (−) and after (+) the addition of NaI.

## 3. Materials and Methods

### 3.1. D-QSAR Pharmacophore Modeling and Virtual Screening

The procedure has been reported in the literature [54,66,67,68,69,70].

### 3.2. Chemistry

All reagents and solvents were purchased from commercial sources. All synthesized compounds were first purified by silica flash chromatography and analyzed by IR, NMR, and HPLC/MS, assessing a purity grade (>95%). Melting points were determined by Kofler. ^1^H NMR and ^13^C NMR spectra were registered on a Bruker 300 Avance (300 MHz) spectrometer, with TMS as an internal standard. IR spectra were recorded by a Shimadzu FTIR-8300 instrument (Shimadzu Corporation, Kyoto, Japan). Chromatography for purification of the samples was realized by flash silica gel (Merck, 0.040–0.063 mm) and mixtures of petroleum ether (fraction boiling at 40–60 °C) and ethyl acetate as eluents. HRMS spectra were recorded by analyzing a 10 ppm solution of each sample in a 6540 UHD Accurate-Mass Q-TOF LC/MS (Agilent Technologies, Inc., Santa Clara, CA, USA) equipped with a Dual AJS ESI source.

#### 3.2.1. Synthesis of NV848 (*N*-(5-methyl-1,2,4-oxadiazol-3-yl)acetamide)

The precursor 3-amino-5-methyl-1,2,4-oxadiazole was prepared as follows: 9.5 g (0.22 mmol) of cyanamide (previously purified with diethyl ether) was added to 1.47 g (0.22 mmol) of hydroxylamine hydrochloride solubilized in EtOH absolute. The reaction was refluxed for 8 h. After this time, the solvent was removed under vacuum, and 30 g (0.31 mol) of potassium acetate and 50 mL of acetic anhydride (0.53 mol) were added in three portions to the product, cooling the bottom flask with an ice bath. Then, the white solution was refluxed for 30 min. After cooling, the reaction was decomposed with 300 mL of ice and water. The solution was alkalinized with NaOH (45 g) and maintained at 80 °C (bain-marie) for 45 min. In the end, the reaction was extracted with diethyl ether (6 × 100 mL).

Reaction yield 36%, MP 119–121 °C. IR (cm^−1^): 3379, 3316, 3183. ^1^H NMR (DMSO-*d*_6_): 2.43 (s, 3H), 6.16 (s, 2H).

Additionally, 600 mg of 3-amino-5-methyl-1,2,4-oxadiazole was solubilized into 1 mL of pyridine and 3 mL of acetic anhydride. The reaction was allowed to sit at room temperature overnight. After this time, the reaction was treated with water and extracted with ethyl acetate. The product was purified by chromatography.

**NV848**: Reaction yield 50%, MP 162–163 °C. IR (cm^−1^): 3254, 3205, 3120, 1695. ^1^H NMR (DMSO-*d*6) δ (ppm): 2.15 (s, 3H), 2.60 (s, 3H), 10.86 (s, 1H).

#### 3.2.2. Synthesis of NV930 (ethyl 5-phenyl-1,2,4-oxadiazole-3-carboxylate)

**NV930** was prepared by the classic amidoxime route: The amidoxime **1** (0.3 g) [71] in toluene (50 mL) was placed in a 250 mL round-bottomed flask. After the addition of 1.2 eq. of benzoyl chloride and 1.2 eq. of pyridine, the reaction mixture was refluxed for 6–8 h, monitoring the consumption of starting material by TLC. The solvent was removed under reduced pressure, followed by water addition and extraction with ethyl acetate. The extracted reaction mixture was chromatographed on silica gel, using eluent mixtures of petroleum ether and ethyl acetate. The obtained sample of the oxadiazole **NV930** was further purified by crystallization in ethanol.

**NV930**: Reaction yield 80%, MP 50–52 °C, from ethanol. ^1^H NMR (300 MHz, CDCl_3_) δ (ppm): 8.22 (d, *J* = 7.2 Hz, 2H), 7.64 (t, *J* = 7.4 Hz, 1H), 7.55 (t, *J* = 7.4 Hz, 2H), 4.55 (q, *J* = 7.1 Hz, 2H), 1.47 (t, *J* = 7.1 Hz, 3H). FT-IR (cm^−1^), 1720. HRMS for C_11_H_10_N_2_O_3_ found 219.0699 [M + H]^+^ (Calcd. 219.0691).

#### 3.2.3. Synthesis of NV914 (2,3,4,5,6-pentafluoro-*N*-(5-(perfluorophenyl)-1,2,4-oxadiazol-3-yl)benzamide)

**NV914** was prepared by acylation with perfluorobenzoyl chloride of the amine **2** [72,73,74].

1.2 eq. of perfluorobenzoyl chloride and 1.2 eq. of pyridine were added to 1.0 eq of the amine **2** dissolved in toluene, and the reaction mixture was refluxed for 4 h, monitoring the consumption of the starting material by TLC. Solvent was removed under reduced pressure followed by water addition and extraction with ethyl acetate. The extracted reaction mixture was chromatographed on silica gel using eluent mixtures of petroleum ether and ethyl acetate. The sample was further purified by crystallization in ethanol.

**NV914**: Reaction yield 90%, MP 208–210 °C, from ethanol. ^1^H NMR (300 MHz, CDCl_3_) δ (ppm): 9.09 (s, 1H), FT-IR (cm^−1^), 3290, 3180, 1750. HRMS for C_15_HF_10_N_3_O_2_ found 445.9917 [M + H]^+^ (Calcd. 445.9909).

### 3.3. Cell Culture Conditions and Transfection of Reporter Plasmid

HeLa cells were cultured in a humidified atmosphere of 5% CO_2_ in air at 37 °C in DMEM medium supplemented with FBS 10% (GIBCO).

Fisher rat thyroid (FRT) cells were cultured in 6-well plates with Coon’s modified F12 medium and the addition of fetal bovine serum (10% FBS), 2.68 g/L sodium bicarbonate, 2 mM glutamine, 100 U/mL penicillin, and 100 μg/mL streptomycin. Antibiotics were removed before the addition of TRIDs.

### 3.4. Measurement of Luciferase Activity by Luminescence

HeLa cells were transfected with Fluc-WT and Fluc-opal plasmids combined with lipofectamine 2000 (Invitrogen) in a 6-well plate at a density of 2 × 10^5^ /ml Cells were incubated for 24 h, then PTC124 and the three selected molecules (12 μM) were added for an additional 24 h. After washing with PBS, cells were incubated with the detection mix Steady-Glo luciferase reagent (Promega, Madison, WI, USA). Then, 200 μL of cell suspension was plated in triplicate in a 96-well plate. Luciferase activity was finally measured on a luminometer (Promega, Madison, WI, USA).

### 3.5. Immunofluorescence Microscopy

Cells grown on rounded glass coverslips were fixed with methanol for 2 min. The wheat germ agglutinin (WGA) Alexa 594 (Life Technologies, Carlsbad, CA, USA) was used to stain the cell membrane and Golgi apparatus, while nuclei were visualized with DAPI. CFTR was detected by incubation with a mouse monoclonal antibody (ab570-mouse 1:500) overnight at 4 °C, followed by a goat polyclonal to mouse Alexa-Fluor-488 (Abcam, 1:1000, Cambridge, UK) secondary antibody for 1 h at 37 °C. Cells were observed under a Zeiss Axioskop microscope (Oberkochen, Baden-Württemberg, Germany) equipped for fluorescence.

### 3.6. Western Blotting

Proteins (30 μg) separated by 10% SDS-PAGE and 3–8% SDS-PAGE (0.1% SDS) were transferred to Hybond-C nitrocellulose membranes (Life Technologies, Carlsbad, CA, USA by electroblotting. The blot was incubated with a mouse antibody anti–CFTR (Ab570 1:500), and HRP-conjugated anti-mouse (PIERCE, 1:5000). The target protein was detected by ECL reagents (Pierce -Thermo Fisher Scientific, Rodano, Italy)). Anti-β-tubulin antibody (mouse; Sigma-Aldrich, St. Louis, MO, USA, 1:10,000) confirmed the presence of an equal amount of proteins per well. Gel bands were quantified by ImageJ software.

### 3.7. Microscope Fluorescence YFP Assay

FRT cells expressing the halide-sensitive YFP and the human WT-CFTR, G542X-CFTR, or W1282X-CFTR cDNA were plated in a 96-well plate. After 24 h, the cells were treated with the test compounds for 24 h. Cells were then washed with phosphate-buffered saline (PBS: 137 mM NaCl, 2.7 mM KCl, 8.1 mM Na_2_HPO_4_, 1.5 mM KH_2_PO_4_, 1 mM CaCl_2_, and 0.5 mM MgCl_2_) and prestimulated for 20 min with forskolin (20 μM, acute dose; Selleckem, Houston, TX, USA) in a final volume of 60 μL. Microplates were then transferred to a fluorescence microscope (ZEISS, Oberkochen, Baden-Württemberg, Germany). YFP fluorescence images were captured by Axiocam ZEISS before (t^0^) and 30 s (t^30s^) after the addition of 165 μL (100 mM/well) of PBS-NaI, an iodide-containing solution. All images were captured with exposition time fixed at 800 ms.

### 3.8. Microplate Reader Fluorescence YFP Assay

FRT cells expressing the human WT-CFTR, G542X-CFTR cDNA, or W1282X-CFTR cDNA, together with the halide-sensitive YFP, were plated in a-96 well plate and, after 24 h, treated with the test compounds. After 24 h of treatment, the cells were washed with phosphate-buffered saline (PBS: 137 mM NaCl, 2.7 mM KCl, 8.1 mM Na_2_HPO_4_, 1.5 mM KH_2_PO_4_, 1 mM CaCl_2_, and 0.5 mM MgCl_2_) and prestimulated for 20 min with forskolin (20 μM, acute dose; Selleckem) in a final volume of 60 μL in PBS1X. Microplates were then transferred to a microplate reader to measure the fluorescence before (t^0^) and 30 s (t^30s^) after the addition of 165 μL (100 mM/well) of PBS-NaI, an iodide-containing solution. The assay was performed in triplicate.

### 3.9. Data Analysis

All data are expressed as mean values ± standard error of the mean. Statistical analysis was performed by Student’s *t*-test and one-way ANOVA followed by Dunnet’s posthoc test, when appropriate (by GraphPad Prism Software version 8.0.0 for Windows, San Diego, CA, USA). A probability value (*p*) of less than 0.05 was regarded as significant and indicated in relevant graphs as one symbol (*) for *p* < 0.05, two symbols (**) for *p* < 0.01, and three symbols (***) for *p* < 0.001.

## 4. Conclusions

The readthrough approach to nonsense is worth being considered as therapy for genetic disorders caused by the presence of a PTC in the mRNA.

The identification of molecules displaying readthrough activity of the PTCs is a very hot topic in the challenge posed by nonsense mutations to find a valuable therapeutic treatment for the second major cause of CF.

Some pitfalls in the readthrough process, when known, can be faced. One issue is that the NMD pathway may lower the efficacy of TRIDs and, in this case, NMD inhibition associated with TRID treatment can improve the amount of rescued protein.

In some cases, TRIDs can revert a nonsense mutation to a missense mutation, thus producing a nonfunctional protein. In these situations, associating appropriate drugs to correct the missense mutation effect should be considered as a possible solution. For this reason, in the search for new TRIDs, besides quantitatively testing the amount of protein expressed by the induced readthrough, it is of paramount importance to also test the functional recovery.

In this project, we designed, synthesized, and biologically tested three new 1,2,4-oxadiazoles TRIDs showing a high readthrough activity with the production of a full length, and most importantly, functional CFTR protein from mutant CFTR cDNAs carrying opal stop mutations.

All three molecules evidenced higher activity than ataluren when tested at 12 μM, the concentration at which ataluren shows its best response, and even higher activity at 24 μM, as evidenced in the dose–response analysis.

These molecules show high readthrough efficacy, both in terms of protein recovery, as confirmed by WB analysis, and in terms of protein functionality. Following the treatment with the new compounds, we observed Fluc-opal luminescence recovery in HeLa cells. Then, by immunofluorescence, in FRT cells engineered with mutant CFTR cDNAs, we observed that CFTR rescued protein was properly localized on the cell membrane. Interestingly, by halide-sensitive YFP-based assay, we demonstrated that TRIDs restored the function of the CFTR channel encoded by PTCs harboring mRNAs. Moreover, the three molecules showed lower cytotoxic effects when compared to ataluren and the previously reported NV2445.

Notwithstanding their limits, pharmacological approaches to nonsense suppression are a fascinating field in view of a wider application to different genetic diseases whose common denominator is a nonsense mutation. In this context, the various processes involved in the readthrough pathway require future research on synergistic effects of NMD inhibition, readthrough induction, and postreadthrough correction.

## 5. Patents

I. Pibiri, A. Pace, M. Tutone, L. Lentini, R. Melfi, A. Di Leonardo, Oxadiazole Derivatives For The Treatment Of Genetic Diseases Due To Nonsense Mutations, PCT Int. Appl. (2019), WO 2019/101709 A1 20190531.

## Supplementary Materials

The following are available online at https://www.mdpi.com/1422-0067/21/17/6420/s1.

## Abbreviations

TRID	Translational Readthrough Inducing Drugs
CFTR	Cystic Fibrosis Transmembrane Regulator
CF	Cystic Fibrosis
Ns	Nonsense
NMD	Nonsense Mediated Decay
PTC	Premature Termination Codon
YFP	Yellow Fluorescent Protein
DMD	Duchenne Muscular Dystrophy
FRT	Fischer Rat Thyroid
DAPI	4′,6-diamidino-2-phenylindole
WB	Western Blot
