# Supplementary material for: Targeting Nonsense: Optimization of 1,2,4-Oxadiazole TRIDs to Rescue CFTR Expression and Functionality in Cystic Fibrosis Cell Model Systems
Source: Int J Mol Sci. 2020 Sep 3;21(17):6420. doi: 10.3390/ijms21176420 (PMC7504161; doi:10.3390/ijms21176420)
Supplement: Supplementary file 1 [file ijms-21-06420-s001.pdf]

International Journal of *Molecular Sciences*

MDPI

Supplementary Material

Targeting Nonsense: Optimization of 1,2,4-Oxadiazole TRIDs to Rescue CFTR Expression and Functionality in Cystic Fibrosis Cell Model Systems

Ivana Pibiri [1,*], Raffaella Melfi [1], Marco Tutone [1], Aldo Di Leonardo [1,2], Andrea Pace [1] and Laura Lentini [1,*]

1 Dipartimento di Scienze e Tecnologie Biologiche, Chimiche e Farmaceutiche (STEBICEF), Università degli Studi di Palermo, Viale delle Scienze Ed. 16-17, 90128 Palermo, Italy; raffaella.melfi@unipa.it (R.M.); marco.tutone@unipa.it (M.T.); aldo.dileonardo@unipa.it (A.D.L.); andrea.pace@unipa.it (A.P.)

2 Centro di OncoBiologia Sperimentale (COBS), via San Lorenzo Colli, 90145 Palermo, Italy

* Correspondence: ivana.pibiri@unipa.it (I.P.); laura.lentini@unipa.it (L.L.); Tel.: +39-091-238-97545 (I.P.); +39-091-238-97341 (L.L.)

1. Testing cell proliferation and viability

The cell viability was analyzed by the MTT assay after 24 and 72 hours of exposition to PTC124, NV2445, **NV848**, **NV930**, and **NV914**. The assay showed that cell viability was already affected by PTC124 (48µM) at 24 hours of treatment. NV2445 showed the worst performance in terms of safety profile. In contrast **NV848**, **NV914**, and **NV930** molecules did not show altered cell viability or proliferation (Figure S1) at the same time points. A partial cytotoxic effect was observed at 72 hours, especially after the addition of **NV930** at the doses of 24 and 48µM (Figure S1).

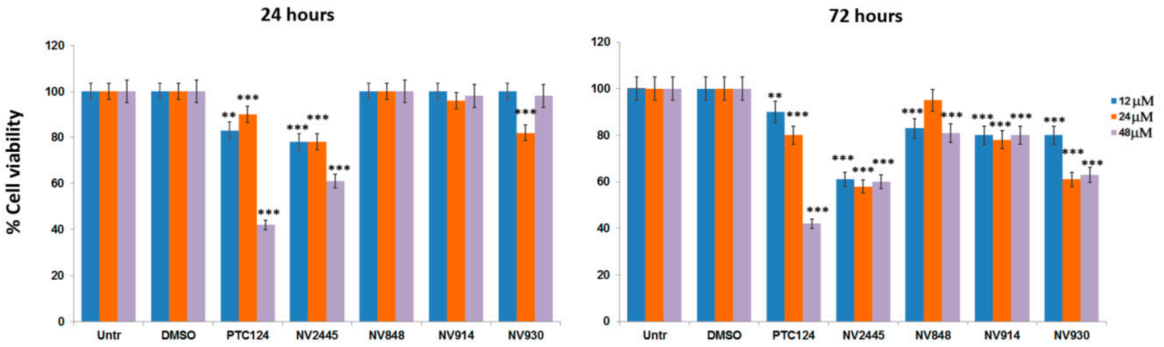

Figure S1. MTT assay in FRT cells to evaluate cell viability after 24-72 hours of treatment with high concentrations (12, 24, 48 µM) of NV selected molecules compared to untreated, DMSO and PTC124. Data were analyzed by GraphPad Prism 6 software and expressed as mean values ± standard error of the mean (S.E.M.). Symbol (*) represent statistical significance of PTC124, **NV848**, **NV914**, and **NV930** versus Untr.: *, $p < 0.05$; **, $p < 0.01$; ***, $p < 0.001$ (ANOVA with Dunnett's post hoc test).

2. Dose-response activity

We analyzed the dose/response activity of **NV848**, **NV914**, and **NV930** at 3, 6, 12, 24, and 48 µM doses. As shown in Figure S2, CFTR expression was detected at all tested concentration and 24 µM was the concentration at which the higher CFTR expression was observed for **NV848** and **NV930**. **NV914** showed a slightly higher activity at 48 than at 24 µM doses. EC50 of **NV848** and **NV914** was calculated by GraphPad Prism 6 software and reported in table S1, while for **NV930** data fitting was not satisfactory, although a rough estimate of EC50 = 13 µM could be calculated based on the maximum activity recorded at the 24 µM dose [1].

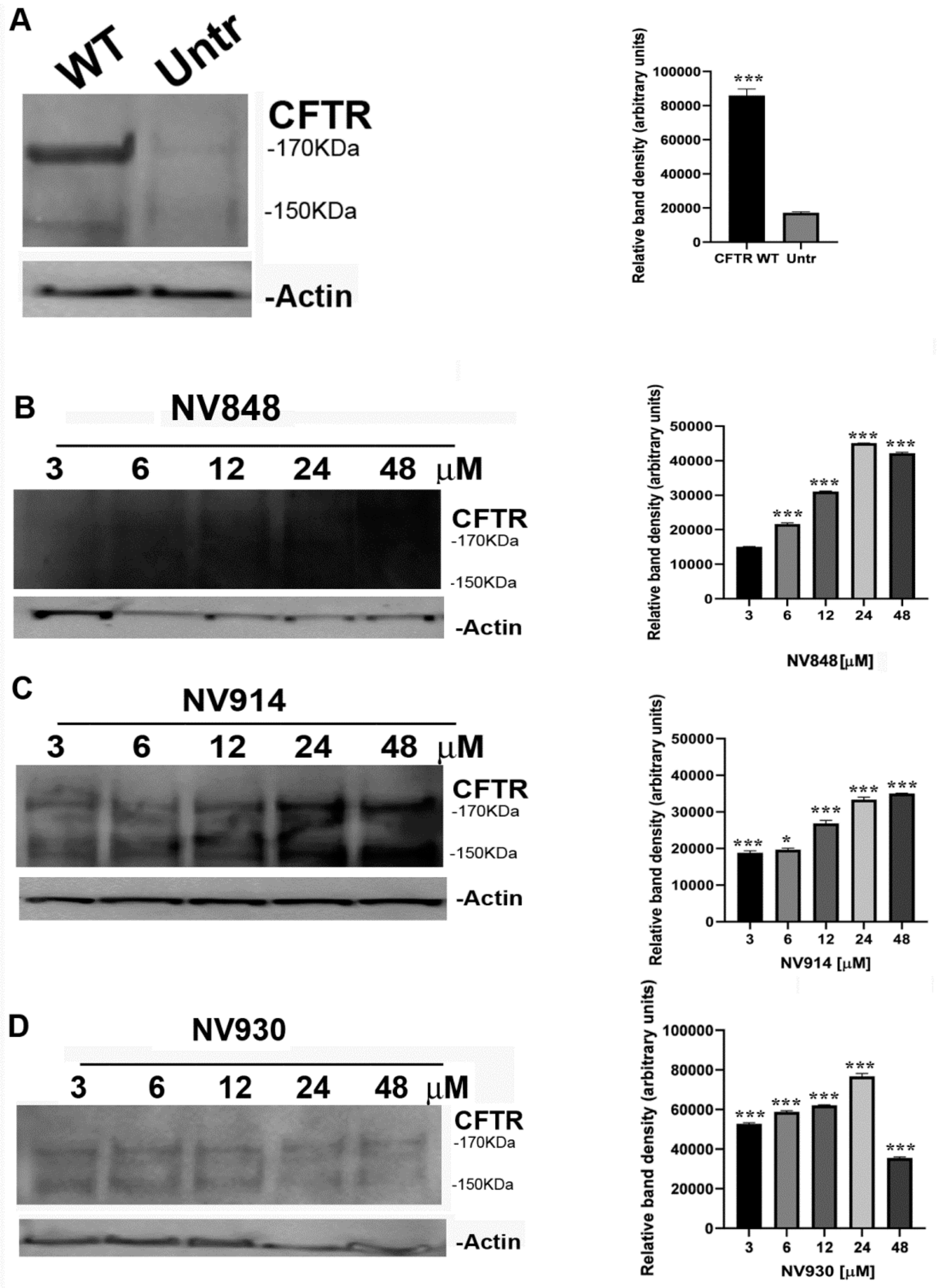

Figure S2. Dose/response measurement after treatment with five different concentrations of **NV848**, **NV914**, and **NV930** in FRT CFTR^{W1282X} cells. Western blot analysis revealed CFTR expression after 24 h of treatment with 3, 6, 12, 24, and 48 μM of **NV848**, **NV914**, and **NV930**. Graphs on the right show relative band density measured by ImageJ software. Data were analyzed by GraphPad Prism 6 software and expressed as mean values ± standard error of the mean (S.E.M.). Symbol (*) represent statistical significance of PTC124, **NV848**, **NV914** and **NV930** versus Untr.: *, $p < 0.05$; **, $p < 0.01$; ***, $p < 0.001$ (ANOVA with Dunnett's post hoc test).

Table 1. EC50 in FRT CFTR^{W1282X} cells.

**NV848**	6.5 μM	95%CL 0.02μM - 1.8mM
**NV914**	16.4 μM	95%CL 0.3μM - 810 μM

3. Quantification of the CFTR expression by fluorescence measure with ImageJ software

Quantification of CFTR expression in Figure 6 and 7 of the manuscript was done manually by using ImageJ software. The background was subtracted and the integrated signal intensity in the selected area (one cell) was measured.

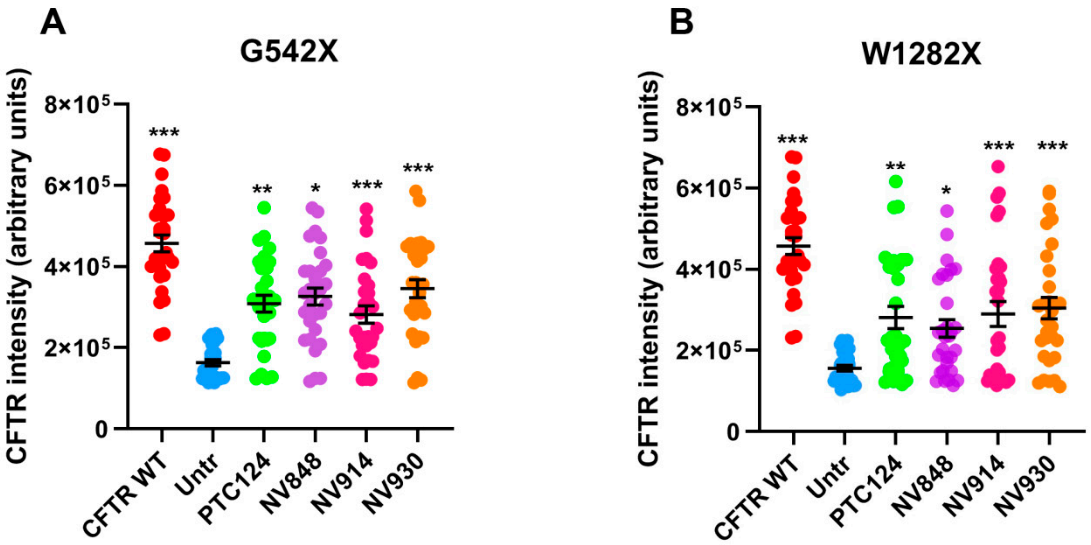

Figure S3. Quantitation of the CFTR signal in immunofluorescence analysis shown in Figure 6 and 7. Graphs show CFTR intensity measured in immunofluorescence analysis in FRT $CFTR^{WT}$ and FRT $CFTR^{G542X}$ and $CFTR^{W1282X}$. Data were analyzed by GraphPad Prism 6 software and expressed as mean values ± standard error of the mean (S.E.M.). Symbol (*) represents statistical significance of **PTC124**, **NV848**, **NV914**, and **NV930** versus Untr.: *, $p < 0.05$; **, $p < 0.01$; ***, $p < 0.001$ (ANOVA with Dunnett's post hoc test).

References

[1] Alexander, B.; Browse, D.J.; Reading, S.J.; Benjamin, A simple and accurate mathematical method for calculation of the EC50. I.S. *Pharmacol Toxicol* **1999**, 41, 55–58.
